# Preoperative Prediction of Total Small Bowel Length Using Anthropometric Measurements: Development and Internal Validation of a Model in 327 Adult Autopsies

**DOI:** 10.3390/diagnostics16182955

**Published:** 2026-09-13

**Authors:** Sinan Şener, Fatih Türkoğlu, Mehmet Fatih Öztürk, Akın Çalışır, Ahmet Depreli, Kamil Hakan Doğan

**Affiliations:** 1 Department of General Surgery, Selçuk University Faculty of Medicine, 42130 Konya, Türkiye; drfatihturkoglu@hotmail.com (F.T.); drmehmetfatihozturk@gmail.com (M.F.Ö.); dracalisir@gmail.com (A.Ç.); 2 Council of Forensic Medicine, Konya Group Presidency, 42130 Konya, Türkiye; ahmetdep@gmail.com; 3 Department of Forensic Medicine, Selçuk University Faculty of Medicine, 42130 Konya, Türkiye; drhakan2000@gmail.com

**Keywords:** small bowel length, anthropometry, bariatric surgery, prediction model, autopsy

## Abstract

**Background/Objectives**: Total small bowel length (TSBL) exhibits considerable interindividual variability and may influence outcomes following bariatric and metabolic surgery. This study aimed to evaluate the relationship between anthropometric characteristics and TSBL and to develop and internally validate an anthropometry-based prediction model. **Methods**: In this prospective observational study, 327 adult autopsy cases were included. Height, weight, body mass index, waist circumference, and abdominal fat thickness were recorded using a standardized protocol. TSBL was measured from the ligament of Treitz to the ileocecal junction. Correlation analyses, multivariable linear regression, and prediction model development were performed. Internal validation was conducted using 1000 bootstrap resamples. Model performance was additionally assessed using prediction error, calibration, and agreement between predicted and observed TSBL. **Results**: Mean TSBL was 612.4 ± 108.7 cm. Height showed the strongest correlation with TSBL (r = 0.612, *p* < 0.001), followed by weight (r = 0.428, *p* < 0.001) and waist circumference (r = 0.341, *p* < 0.001). In multivariable analysis, height (standardized β = 0.447, *p* < 0.001), weight (standardized β = 0.185, *p* = 0.006), and waist circumference (standardized β = 0.099, *p* = 0.029) remained independently associated with TSBL. The final prediction model yielded R^2^ = 0.456 and adjusted R^2^ = 0.451, with a root mean square error of 80.1 cm and mean absolute error of 62.8 cm. Bootstrap validation yielded an optimism-corrected R^2^ of 0.444 and a calibration slope of 0.97. Predicted TSBL was within ±10% of the observed value in 56.0% of cases. **Conclusions**: Height was the strongest independent predictor of TSBL, while weight and waist circumference provided additional predictive information. The proposed anthropometry-based model showed moderate predictive performance but retained substantial individual prediction error. It should therefore be considered an exploratory tool that may provide supplementary preoperative information rather than a substitute for direct bowel measurement. External validation in living and independent populations is required before clinical implementation.

## 1. Introduction

Obesity is a chronic and multifactorial disease that has reached epidemic proportions worldwide and is associated with substantial morbidity, mortality, and healthcare expenditure. Bariatric and metabolic surgery has become the most effective long-term treatment for severe obesity and obesity-related metabolic disorders, providing superior and more durable outcomes compared with lifestyle modification and pharmacological interventions. As the number of bariatric procedures continues to increase globally, there is growing interest in identifying patient-specific anatomical and physiological factors that may influence surgical outcomes and facilitate individualized operative planning [1,2,3].

Among these factors, total small bowel length (TSBL) has attracted considerable attention because of its direct relevance to nutrient absorption and postoperative metabolic outcomes. In malabsorptive and mixed bariatric procedures, including Roux-en-Y gastric bypass, one-anastomosis gastric bypass, and biliopancreatic diversion with duodenal switch, the configuration and length of intestinal limbs play a critical role in determining the balance between weight loss efficacy and the risk of nutritional complications. Previous studies have demonstrated substantial interindividual variation in TSBL, suggesting that a standardized surgical approach may not be equally appropriate for all patients. Consequently, there is increasing recognition that a more individualized strategy, incorporating patient-specific intestinal anatomy, may improve both surgical precision and postoperative outcomes [4,5,6]. Knowledge of TSBL before surgery could therefore provide additional anatomical information when selecting a bariatric procedure and planning alimentary, biliopancreatic, or common-channel lengths. In particular, an estimate of the patient’s overall bowel length could help place a proposed limb configuration within the context of individual intestinal anatomy rather than relying exclusively on fixed limb lengths.

Despite its potential clinical importance, TSBL is rarely known before surgery and is typically determined through intraoperative bowel measurement. This process requires manual assessment of the small intestine and may prolong operative and anesthesia times, particularly in technically demanding cases. Extended operative duration has been associated with an increased risk of perioperative complications, including thromboembolic events, pulmonary complications, and delayed recovery [5,7]. A preoperative estimate of TSBL would not necessarily replace direct intraoperative measurement when precise anatomical determination is required, but it could provide supplementary information before surgery and support individualized operative planning. Several investigators have explored possible associations between TSBL and anthropometric characteristics such as height, body weight, body mass index, and waist circumference. However, the reported findings have been inconsistent, and the available evidence remains limited by relatively small sample sizes, heterogeneous measurement methodologies, and the predominance of intraoperative rather than anatomical investigations. Furthermore, clinically applicable prediction models capable of estimating TSBL with acceptable accuracy have not yet been adequately established [8,9]. Thus, the principal knowledge gap is not simply whether anthropometric characteristics are associated with bowel length, but whether routinely obtainable preoperative variables can be combined into a reproducible prediction model and how much of the marked interindividual variability in TSBL such a model can realistically explain. Prediction-model development also requires assessment beyond statistical associations alone. Model performance, prediction error, calibration, and internal validation are necessary to determine whether an equation derived from anatomical and anthropometric relationships has sufficient stability to warrant further clinical investigation. In this context, a relatively large standardized anatomical dataset may provide a useful framework for developing an exploratory preoperative prediction model.

Postmortem investigations provide a unique opportunity to obtain standardized anatomical measurements of the gastrointestinal tract and to explore the relationship between anthropometric characteristics and intestinal morphology. Compared with intraoperative measurements, autopsy-based assessment permits bowel measurement under more standardized conditions, without constraints related to operative exposure, pneumoperitoneum, patient positioning, or the need to minimize operative time. At the same time, postmortem measurements may be affected by loss of physiological tone and tissue relaxation, and therefore require cautious interpretation when extrapolated to living surgical populations. Large-scale autopsy-based studies evaluating the association between anthropometric measurements and TSBL remain limited, and clinically applicable prediction models have not been adequately investigated.

Accordingly, this study was designed to extend previous association-based investigations by combining standardized postmortem anatomical measurements with multivariable prediction-model development and internal validation in a comparatively large autopsy sample. Therefore, the present study aimed to evaluate the relationship between anthropometric measurements and total small bowel length in a large autopsy cohort and to develop and internally validate an anthropometry-based prediction model for estimating TSBL.

## 2. Materials and Methods

### 2.1. Study Design, Setting, and Ethical Approval

This prospective observational study was conducted at the Konya Regional Branch of the Council of Forensic Medicine, Türkiye. The study aimed to evaluate the relationship between anthropometric measurements and total small bowel length in adult autopsy cases and to develop a prediction model for estimating total small bowel length using readily obtainable anthropometric parameters. The study protocol was approved by the Non-Interventional Clinical Research Ethics Committee of Selçuk University Faculty of Medicine (approval number: 2025/703; date: 2 December 2025). In addition, institutional permission to conduct the study was obtained from the Konya Regional Branch of the Council of Forensic Medicine (approval number: 21589509-120.25-L1456; date: 11 November 2025). All study procedures were performed in accordance with the ethical principles of the Declaration of Helsinki and relevant national regulations governing scientific research involving postmortem investigations. Case inclusion and data collection were performed prospectively during routine forensic autopsy examinations. Anthropometric and morphometric measurements were obtained according to a predefined standardized protocol by trained investigators. The study was designed and reported in accordance with the Strengthening the Reporting of Observational Studies in Epidemiology (STROBE) statement.

### 2.2. Study Population

Adult autopsy cases examined at the Konya Regional Branch of the Council of Forensic Medicine during the study period were consecutively screened for eligibility. Cases aged 18 years or older and undergoing routine forensic autopsy were considered for inclusion. To ensure accurate anatomical assessment of the gastrointestinal tract, only cases with preserved intestinal continuity and adequate postmortem tissue integrity were included. Autopsies performed more than 36 h after documented death were not included, and cases showing advanced decomposition were excluded irrespective of the recorded postmortem interval. Cases were excluded if they had a history of previous intestinal resection, major abdominal surgery affecting bowel anatomy, traumatic or pathological disruption of the gastrointestinal tract, advanced postmortem decomposition, incomplete key anthropometric or intestinal measurements, or any condition that could compromise measurement reliability. Cases requiring special isolation procedures because of highly contagious infectious diseases were also excluded for biosafety reasons.

**Figure 1:**
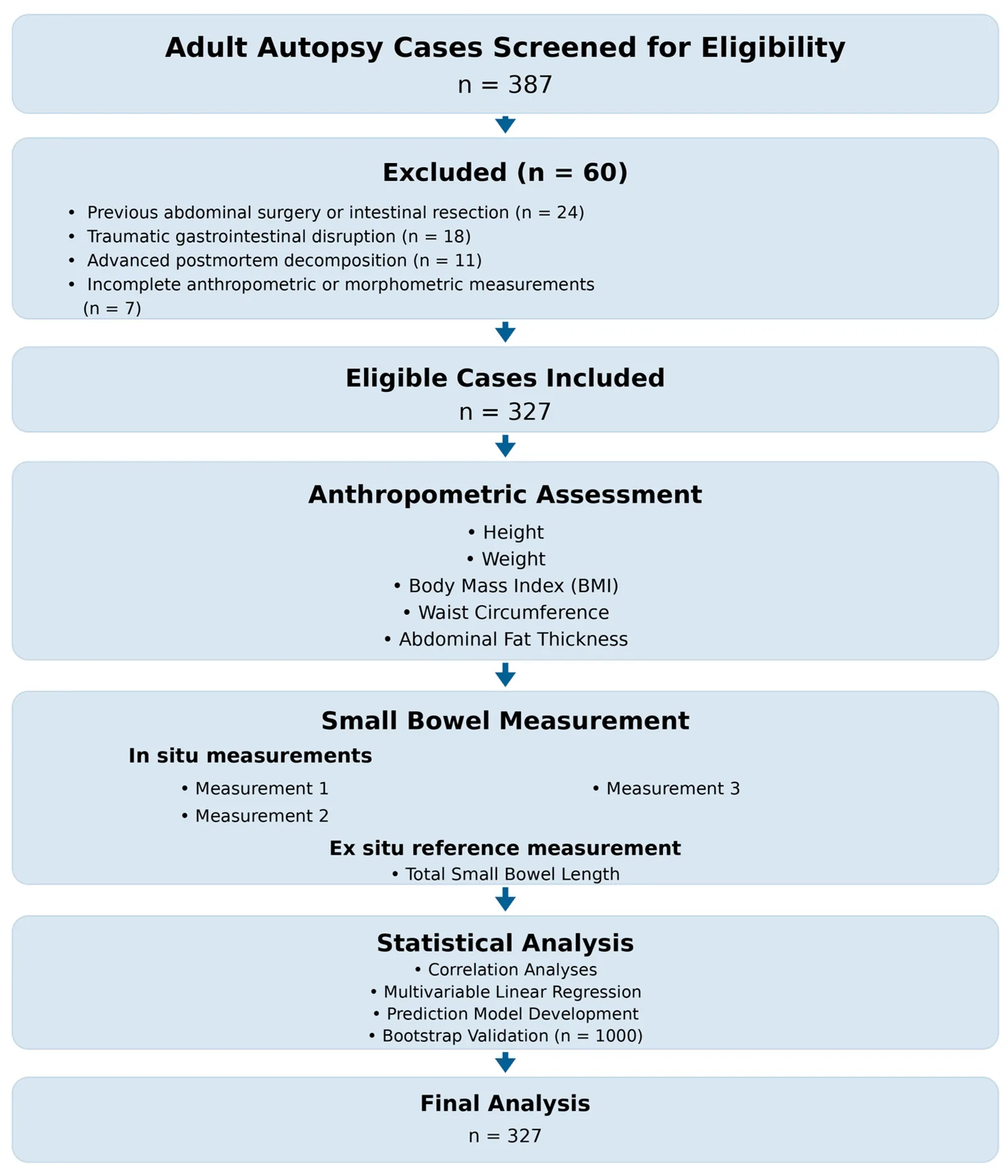
Flow Diagram of Case Selection and Study Inclusion Process.

During the study period, 387 autopsy cases were screened for eligibility. Sixty cases were excluded because of previous major abdominal surgery or intestinal resection (*n* = 16), traumatic or pathological disruption of the small bowel (*n* = 13), advanced postmortem decomposition (*n* = 11), incomplete anthropometric or intestinal measurements (*n* = 12), or other prespecified eligibility criteria (*n* = 8). Consequently, 327 cases were included in the final analysis. The case-selection process and reasons for exclusion are summarized in Figure 1.

### 2.3. Anthropometric Measurements

Anthropometric measurements were obtained during routine forensic autopsy examinations according to a standardized measurement protocol. All measurements were performed by trained investigators before gastrointestinal dissection. Body height was measured in the supine position using a calibrated measuring tape and recorded to the nearest 0.1 cm. Body weight was obtained from routine autopsy records and recorded to the nearest 0.1 kg. Body mass index (BMI) was calculated as weight in kilograms divided by height in meters squared (kg/m^2^). Waist circumference was measured at the midpoint between the lowest palpable rib and the iliac crest using a non-elastic measuring tape and recorded to the nearest 0.1 cm. Abdominal fat thickness was measured during autopsy as the maximal thickness of the anterior abdominal subcutaneous adipose tissue at the midline level and recorded in centimeters.

All anthropometric measurements were performed before bowel manipulation and documented prospectively in an electronic database. To minimize measurement variability, the same measurement techniques and anatomical landmarks were used throughout the study period. The interval between documented death and initiation of the autopsy examination was recorded in hours and evaluated in relation to TSBL, body weight, waist circumference, and abdominal fat thickness to assess potential time-related postmortem effects.

### 2.4. Small Bowel Length Assessment

Total small bowel length was assessed using a predefined standardized protocol. The small intestine was identified from the ligament of Treitz to the ileocecal junction. Measurements were performed both in situ and ex situ.

For in situ assessment, the bowel was measured within the abdominal cavity before complete dissection. Three consecutive measurements were obtained along the antimesenteric border using a flexible measuring tape while avoiding excessive traction or stretching. For each case, all three consecutive in situ measurements were performed by the same investigator using the same technique. These repeated measurements were used to assess measurement reproducibility.

Following the in situ assessment, the entire small intestine was dissected from the ligament of Treitz to the ileocecal junction. Ex situ measurement was performed along the antimesenteric border with the bowel placed on a flat surface under standardized conditions. Excessive tension, compression, or artificial elongation was avoided. Ex situ TSBL was considered the reference anatomical measurement and was used in all primary correlation, regression, and prediction-model analyses. All measurements were performed by investigators experienced in gastrointestinal anatomical assessment using the same standardized technique throughout the study period. Measurements were recorded prospectively in a dedicated electronic database immediately after completion of each autopsy examination.

### 2.5. Data Collection and Study Variables

Demographic, anthropometric, and postmortem data were collected prospectively using a standardized data collection form. Demographic variables included age and sex. Postmortem variables included cause of death and the interval between death and autopsy. Anthropometric variables included height, weight, BMI, waist circumference, and abdominal fat thickness. Morphometric variables included the three consecutive in situ bowel-length measurements and the reference ex situ TSBL measurement. Race/skin color was not routinely recorded in the forensic records and was therefore unavailable for analysis. The primary outcome was ex situ TSBL. Secondary analyses included associations between anthropometric variables and TSBL, identification of independent predictors, development and internal validation of an anthropometry-based prediction model, assessment of repeated-measure reproducibility, sex-stratified associations, and individual-level prediction accuracy.

### 2.6. Development of the Prediction Model

A prediction model for TSBL was developed using anthropometric variables that can be readily obtained before surgery. Univariable analyses were first performed to evaluate relationships between TSBL and candidate demographic and anthropometric variables, including age, sex, height, weight, BMI, waist circumference, and abdominal fat thickness.

Variables associated with TSBL and those considered clinically relevant were evaluated in multivariable linear regression analyses. Age and sex were retained in the full explanatory model because of their biological relevance irrespective of univariable statistical significance. Because BMI is mathematically derived from height and weight and may introduce redundancy when these variables are modeled simultaneously, BMI was not included in the principal multivariable model containing height and weight. Prediction-model development followed a hierarchical approach. Height was entered first, followed sequentially by body weight and waist circumference. Performance was evaluated using R^2^, adjusted R^2^, root mean square error (RMSE), and mean absolute error (MAE). The final parsimonious prediction model was selected according to predictive performance and model parsimony, and the prediction equation was derived from its unstandardized regression coefficients. Penalized regression using least absolute shrinkage and selection operator (LASSO) and ridge regression was additionally performed as a sensitivity analysis to assess whether regularization improved predictive performance compared with conventional linear regression.

### 2.7. Internal Validation and Prediction Accuracy

Internal validation of the final prediction model was performed using 1000 bootstrap resamples. Optimism-corrected R^2^, calibration slope, and calibration intercept were calculated.

Agreement between predicted and observed TSBL was assessed using Bland–Altman analysis. Mean prediction bias and 95% limits of agreement were calculated as the mean difference ±1.96 standard deviations of the differences. Proportional bias was assessed by examining the relationship between prediction differences and the corresponding mean values. Individual-level prediction accuracy was additionally expressed as the proportion of cases in which predicted TSBL was within ±10% of the observed ex situ TSBL.

### 2.8. Statistical Analysis

Primary statistical analyses were performed using IBM SPSS Statistics for Windows, version 26.0 (IBM Corp., Armonk, NY, USA). Penalized regression analyses were performed using R software, version 4.4.2 (R Foundation for Statistical Computing, Vienna, Austria), with the glmnet package, version 4.1-8. Continuous variables were assessed for normality using histograms, Q–Q plots, and the Shapiro–Wilk test and were presented as mean ± standard deviation. Categorical variables were reported as *n* (%). Associations between TSBL and continuous variables were evaluated using Pearson correlation analysis. Reproducibility of the three consecutive in situ bowel-length measurements was assessed using a two-way mixed-effects, absolute-agreement, single-measure intraclass correlation coefficient (ICC) with 95% confidence intervals. Mean TSBL was compared between males and females using the independent-samples *t*-test. Height–TSBL correlations were evaluated separately by sex, and a sex × height interaction term was assessed using linear regression. Multivariable linear regression was used to identify independent factors associated with TSBL. Regression assumptions were assessed for linearity, normality of residuals, homoscedasticity, multicollinearity, and influential observations. Multicollinearity was assessed using individual variance inflation factors (VIFs), with VIF < 3.0 considered acceptable. Residual normality and homoscedasticity were evaluated using standard diagnostic procedures, including the Shapiro–Wilk and Breusch–Pagan tests. Residual independence was assessed using the Durbin–Watson statistic, and influential observations were evaluated using Cook’s distance. Prediction-model performance was assessed using R^2^, adjusted R^2^, RMSE, and MAE. Internal validation was performed using 1000 bootstrap resamples, with optimism-corrected R^2^, calibration slope, and calibration intercept reported. Agreement and individual prediction accuracy were evaluated using Bland–Altman analysis and the proportion of predictions within ±10% of observed TSBL. LASSO and ridge regression were evaluated using cross-validated RMSE and compared with the conventional linear regression model. All eligible cases available during the predefined study period were included. With 327 cases and six candidate parameters in the full multivariable model, the dataset provided more than 50 observations per candidate parameter. Model complexity was kept limited, and bootstrap resampling was additionally used to quantify optimism and assess internal model stability. All statistical tests were two-tailed, and *p* < 0.05 was considered statistically significant.

## 3. Results

### 3.1. Study Flow and Baseline Characteristics

A total of 387 adult autopsy cases were assessed for eligibility during the study period. Sixty cases were excluded because of previous major abdominal surgery or intestinal resection (*n* = 16), traumatic or pathological disruption of the small bowel (*n* = 13), advanced postmortem decomposition (*n* = 11), incomplete anthropometric or intestinal measurements (*n* = 12), or other prespecified eligibility criteria (*n* = 8). The final analysis included 327 cases (Figure 1).

The mean age was 44.8 ± 16.7 years, and 238 cases (72.8%) were male. Mean height was 172.6 ± 8.9 cm, mean weight was 79.4 ± 18.6 kg, and mean body mass index (BMI) was 26.5 ± 5.8 kg/m^2^. Mean waist circumference was 94.1 ± 15.2 cm, and mean abdominal fat thickness was 3.8 ± 1.7 cm. The mean ex situ total small bowel length (TSBL) was 612.4 ± 108.7 cm. The mean interval between death and autopsy was 18.7 ± 7.4 h. Baseline demographic, anthropometric, and postmortem characteristics are presented in Table 1.

The intraclass correlation coefficient for the three repeated in situ measurements was 0.987 (95% CI, 0.984–0.990).

The interval between death and autopsy was not significantly correlated with TSBL (r = 0.041, *p* = 0.460), body weight (r = −0.052, *p* = 0.349), waist circumference (r = 0.063, *p* = 0.256), or abdominal fat thickness (r = 0.049, *p* = 0.377).

### 3.2. Correlation Analyses

Height showed the strongest positive correlation with TSBL (r = 0.612, *p* < 0.001), followed by body weight (r = 0.428, *p* < 0.001) and waist circumference (r = 0.341, *p* < 0.001). BMI (r = 0.221, *p* < 0.001) and abdominal fat thickness (r = 0.196, *p* < 0.001) were also positively correlated with TSBL. No statistically significant correlation was observed between age and TSBL (r = −0.062, *p* = 0.264) or between the death-to-autopsy interval and TSBL (r = 0.041, *p* = 0.460) (Table 2).

### 3.3. Sex-Stratified Analyses

Mean TSBL was 623.6 ± 106.8 cm in males and 582.5 ± 108.1 cm in females, corresponding to a mean difference of 41.1 cm (95% CI, 14.9–67.3; *p* = 0.002). Height was positively correlated with TSBL in both males (r = 0.578, *p* < 0.001) and females (r = 0.521, *p* < 0.001). In the interaction analysis, the sex × height interaction term was not statistically significant (*p* = 0.614).

### 3.4. Multivariable Linear Regression Analysis

Age, sex, height, weight, waist circumference, and abdominal fat thickness were included in the multivariable linear regression model. Height (unstandardized β = 5.46; 95% CI, 4.21–6.71; standardized β = 0.447; *p* < 0.001), body weight (unstandardized β = 1.08; 95% CI, 0.31–1.85; standardized β = 0.185; *p* = 0.006), and waist circumference (unstandardized β = 0.71; 95% CI, 0.07–1.35; standardized β = 0.099; *p* = 0.029) were independently associated with TSBL.

Age (*p* = 0.410), sex (*p* = 0.281), and abdominal fat thickness (*p* = 0.314) were not independently associated with TSBL. The model yielded an R^2^ of 0.461 and an adjusted R^2^ of 0.451 (F = 45.6, *p* < 0.001).

Individual variance inflation factor values ranged from 1.05 to 2.54 (Table 3). The Shapiro–Wilk test for regression residuals yielded *p* = 0.081, the Breusch–Pagan test yielded *p* = 0.236, and the Durbin–Watson statistic was 1.96. No observation had a Cook’s distance > 1.0.

### 3.5. Prediction Model Development and Internal Validation

Three sequential prediction models were evaluated. Height alone yielded an R^2^ of 0.374 and an adjusted R^2^ of 0.372, with an RMSE of 85.9 cm and an MAE of 67.7 cm. Addition of body weight increased the R^2^ to 0.439 and the adjusted R^2^ to 0.436, with an RMSE of 81.3 cm and an MAE of 64.1 cm.

The final model included height, body weight, and waist circumference. The model yielded an R^2^ of 0.456, adjusted R^2^ of 0.451, RMSE of 80.1 cm, and MAE of 62.8 cm (Table 4).

The final prediction equation was: Predicted TSBL (cm) = −459.0 + 5.25 × Height (cm) + 1.18 × Weight (kg) + 0.76 × Waist circumference (cm).

Bootstrap internal validation yielded an optimism-corrected R^2^ of 0.444, an estimated optimism of 0.007, a calibration slope of 0.97, and a calibration intercept of 18.4 cm.

In the Bland–Altman analysis, the mean difference between predicted and observed TSBL was 0.2 cm, with 95% limits of agreement from −156.2 to 156.6 cm. No statistically significant proportional bias was identified (*p* = 0.412). Predicted TSBL was within ±10% of the observed value in 183 of 327 cases (56.0%).

### 3.6. Sensitivity Analysis

Penalized regression models were evaluated as sensitivity analyses. LASSO regression retained height, body weight, and waist circumference. The cross-validated RMSE was 80.5 cm for LASSO regression and 80.2 cm for ridge regression, compared with 80.1 cm for the conventional linear regression model.

## 4. Discussion

In this large autopsy-based study, we investigated the relationship between anthropometric characteristics and total small bowel length and developed a prediction model using routinely obtainable clinical measurements. Several noteworthy findings emerged from the analysis. First, considerable interindividual variation in total small bowel length was observed, even within a relatively homogeneous adult population. Second, body height was identified as the strongest determinant of bowel length and remained independently associated with total small bowel length after adjustment for other anthropometric variables. Third, body weight and waist circumference also contributed independently to bowel length variation, whereas age, sex, and abdominal fat thickness did not retain statistical significance in multivariable analyses. Although mean TSBL was greater in males than in females, the sex × height interaction was not significant, and sex did not remain independently associated with TSBL after anthropometric adjustment. Finally, a prediction model incorporating height, weight, and waist circumference explained approximately 46% of TSBL variability; however, an RMSE of approximately 80 cm and predictions within ±10% of the observed TSBL in only 56.0% of cases indicated substantial residual individual-level prediction error. Taken together, these findings indicate that external anthropometric characteristics capture a meaningful, but incomplete, component of individual intestinal anatomy and support further investigation of anthropometry-based preoperative estimation rather than its immediate clinical implementation [8,10]. One of the most notable findings of the present study was the substantial variability in total small bowel length observed among individuals. Although the mean ex situ small bowel length was 612.4 cm, measurements varied considerably across the cohort, highlighting the marked anatomical heterogeneity of the human small intestine. This finding is consistent with previous anatomical and surgical studies reporting wide interindividual differences in bowel length, with published estimates ranging from less than 300 cm to more than 1000 cm depending on the study population and measurement methodology. Such variability suggests that intestinal anatomy is influenced by a complex interplay of genetic, developmental, environmental, and anthropometric factors rather than a single determinant [11].

The clinical implications of this variability are particularly relevant in bariatric and metabolic surgery. In procedures incorporating a malabsorptive component, such as Roux-en-Y gastric bypass, one-anastomosis gastric bypass, and biliopancreatic diversion with duodenal switch, the proportion of bypassed intestine may differ substantially between individuals despite the use of identical limb lengths. Consequently, patients with shorter total bowel lengths may experience greater degrees of malabsorption and nutritional deficiency, whereas those with longer intestines may exhibit reduced metabolic and weight-loss responses following surgery. This concept has contributed to growing interest in individualized surgical planning based on patient-specific intestinal anatomy rather than relying exclusively on fixed limb lengths [12,13]. From this perspective, knowledge of TSBL before surgery could theoretically provide additional anatomical context when considering limb configuration; nevertheless, the precision required for operative decision-making is greater than that demonstrated by the present prediction model.

The mean bowel length identified in our cohort falls within the range reported in previous autopsy and intraoperative studies. Importantly, the use of a standardized measurement protocol, repeated in situ measurements, and a reference ex situ assessment may have contributed to reducing methodological variability, which has historically represented one of the major challenges in studies evaluating intestinal length. The three consecutive in situ measurements showed high reproducibility (ICC = 0.987), supporting the consistency of measurements obtained under the standardized study protocol. Differences in bowel tension, measurement technique, anatomical landmarks, and the distinction between in situ and ex situ assessments have been proposed as important explanations for the heterogeneity reported across the literature. Therefore, the relatively large sample size and standardized anatomical assessment employed in the present study provide additional evidence regarding the distribution of total small bowel length in adults and represent an anatomical contribution complementary to previous predominantly intraoperative investigations [14,15].

At the same time, an important distinction must be made between postmortem and in vivo bowel measurements. Loss of physiological smooth-muscle tone, tissue relaxation, changes in hydration, and the absence of mesenteric and intra-abdominal physiological forces may alter bowel dimensions after death. In the present study, advanced decomposition was excluded, the death-to-autopsy interval was restricted, and no significant association was detected between this interval and TSBL or the principal anthropometric measurements. These findings reduce concern regarding a major time-dependent effect within the studied postmortem interval but cannot establish equivalence between postmortem and intraoperative bowel length. Accordingly, ex situ measurements should be considered standardized anatomical reference measurements rather than direct surrogates of bowel length under living operative conditions.

Among the anthropometric variables evaluated, body height emerged as the strongest predictor of total small bowel length. Height demonstrated the highest correlation coefficient in univariable analysis and remained the most influential independent determinant after adjustment for other anthropometric characteristics. This finding is biologically plausible, as both stature and intestinal length are influenced by common developmental processes occurring during fetal growth and postnatal maturation. Individuals with greater longitudinal body growth may be expected to possess proportionally larger visceral organs and longer intestinal segments, reflecting overall differences in somatic development [14,16]. The observed association between height and small bowel length is also consistent with several previous anatomical investigations that identified stature as one of the most important determinants of intestinal length. However, the strength of this relationship has varied considerably across studies, likely because of differences in sample size, measurement techniques, ethnic composition, and study design. Some investigations have reported only weak associations, whereas others have described moderate correlations similar to those observed in the present cohort. These discrepancies further emphasize the importance of standardized measurement methodologies when evaluating anatomical structures that are highly susceptible to measurement-related variability [17,18].

From a clinical perspective, the relationship between height and bowel length may have implications for preoperative assessment. Height is among the simplest and most reliable anthropometric variables available in routine clinical practice and can be obtained without additional cost or specialized equipment. Although height alone cannot accurately determine total bowel length, the present findings suggest that it provides information regarding underlying intestinal anatomy. Height alone explained more than one-third of the variability in bowel length. However, this also means that most interindividual variability remained unexplained when stature was considered alone, limiting its usefulness as an isolated estimator of TSBL.

Body weight also demonstrated an independent association with total small bowel length after adjustment for other anthropometric variables. Although the magnitude of this relationship was less pronounced than that observed for height, the persistence of statistical significance in the multivariable model suggests that body mass may capture aspects of anatomical variation that are not fully explained by stature alone. This finding is noteworthy because previous studies evaluating the relationship between body weight and intestinal length have yielded inconsistent results, with some reporting positive associations and others identifying little or no independent effect after accounting for body size [8]. Several mechanisms may explain the observed relationship between body weight and bowel length. The small intestine is a metabolically active organ responsible for nutrient digestion and absorption, and adaptive interactions between intestinal morphology and long-term energy balance have been proposed. Individuals with greater body mass may possess a larger absorptive surface area, partly reflecting increased intestinal length and contributing to differences in nutrient handling and metabolic efficiency. Although the observational anatomical design of the present study precludes conclusions regarding causality, the observed association supports the concept that intestinal anatomy and body composition may be interconnected through shared developmental and physiological pathways [19,20].

The independent contribution of body weight is particularly relevant from a clinical standpoint. Unlike height, which primarily reflects skeletal growth, body weight incorporates multiple biological dimensions, including lean body mass, adiposity, and overall nutritional status. The finding that both height and weight independently contributed to the prediction model suggests that bowel length is influenced not only by longitudinal body growth but also by broader aspects of body habitus. Nevertheless, the relatively modest effect size observed in comparison with height indicates that body weight should be considered a complementary predictor rather than a primary determinant of intestinal length. Consistent with this, adding weight to height increased model R^2^ from 0.374 to 0.439, while the subsequent addition of waist circumference produced a smaller incremental increase [21,22].

Waist circumference remained independently associated with total small bowel length in the adjusted analyses, whereas abdominal fat thickness did not retain statistical significance after multivariable adjustment. These findings suggest that measures reflecting overall body habitus may provide more meaningful information regarding intestinal anatomy than localized assessments of subcutaneous adiposity alone. Although both variables are commonly used as indicators of adiposity, they represent distinct biological constructs and may capture different aspects of body composition [23]. Waist circumference is widely recognized as a surrogate marker of central adiposity and visceral fat accumulation. Compared with body mass index, waist circumference may better reflect metabolically active abdominal fat and overall abdominal volume. The independent association observed in the present study may indicate that individuals with larger abdominal dimensions tend to possess greater intra-abdominal organ development, including longer intestinal segments. While the underlying mechanisms remain incompletely understood, this observation is consistent with the concept that gastrointestinal morphology may be influenced not only by longitudinal growth but also by broader patterns of body size and abdominal development [24,25].

In contrast, abdominal fat thickness demonstrated only a weak association with bowel length in univariable analysis and lost significance after adjustment for other anthropometric variables. This finding suggests that the relationship between adiposity and intestinal length may be largely mediated by overall body size rather than the thickness of subcutaneous abdominal fat itself. From a physiological perspective, localized fat deposition is unlikely to directly influence intestinal growth or development, which may explain its limited independent predictive value. The attenuation of the association after adjustment further supports the possibility that abdominal fat thickness shares substantial variance with other anthropometric parameters, particularly body weight and waist circumference [26]. Variables that are simple, reproducible, and routinely available in clinical practice are generally preferred over measurements requiring additional procedural assessment. Accordingly, the final parsimonious model retained height, weight, and waist circumference, all of which can be obtained without invasive assessment [27].

The development of an anthropometry-based prediction model represents an additional objective of the present study. Although previous investigations have reported associations between anthropometric characteristics and intestinal length, few have attempted to translate these observations into a practical preoperative prediction equation. Previous studies have differed substantially in sample size, population characteristics, and bowel measurement techniques, and many were primarily designed to assess correlations rather than to evaluate a multivariable model using calibration and internal validation procedures. The present study therefore extends this literature by combining a relatively large standardized autopsy dataset with multivariable model development, bootstrap validation, prediction-error estimates, and assessment of agreement between predicted and observed values [10,28].

Nevertheless, the performance estimates require cautious interpretation. The final model yielded an R^2^ of 0.456 and an adjusted R^2^ of 0.451, indicating that more than half of the observed variation in TSBL remained unexplained. Moreover, the RMSE was 80.1 cm and MAE was 62.8 cm. Bland–Altman analysis showed wide 95% limits of agreement (−156.2 to 156.6 cm), and only 56.0% of predicted values were within ±10% of the corresponding observed TSBL. Thus, the near-zero mean prediction bias should not be interpreted as high individual-level accuracy, because substantial differences between predicted and observed bowel length remained in individual cases.

Bootstrap validation yielded an optimism-corrected R^2^ of 0.444 and a calibration slope of 0.97, suggesting limited apparent overfitting within the development dataset. However, internal validation assesses model stability within the same source population and does not establish transportability to other populations or clinical settings. Penalized regression approaches were also explored as sensitivity analyses. LASSO and ridge regression produced RMSE values similar to conventional linear regression and did not materially improve predictive performance. This finding suggests that the principal limitation of the model may reflect information not captured by the available anthropometric variables rather than the choice of regression technique alone. The moderate predictive performance observed in the present study is not unexpected given the multifactorial nature of intestinal development. Developmental growth, body size, and other biological determinants are likely to contribute to bowel morphology and may account for part of the variability not captured by routinely available anthropometric measurements. Therefore, explaining approximately 45% of TSBL variability using three readily obtainable anthropometric measurements should be viewed as evidence of a measurable anatomical relationship rather than evidence of sufficient precision for clinical decision-making [29,30].

These findings may be particularly relevant in bariatric and metabolic surgery, where increasing attention is being directed toward individualized operative strategies. Considerable variation in total small bowel length may contribute to the heterogeneous clinical outcomes observed following procedures that incorporate a malabsorptive component. A preoperative prediction model could potentially provide preliminary anatomical information before surgery; however, based on the observed individual prediction error, the present equation cannot currently be considered sufficiently precise to determine intestinal limb lengths or replace direct intraoperative measurement. Its current role should therefore be regarded as exploratory and hypothesis-generating. External validation, particularly in living bariatric surgical populations, is necessary before its potential clinical utility can be determined.

Beyond its potential surgical implications, the autopsy-based design represents a methodological strength of the present study by enabling standardized anatomical assessment outside the time and exposure constraints of an operative setting. At the same time, this feature defines an important translational limitation because postmortem anatomy does not completely reproduce physiological in vivo conditions. Accordingly, the present findings provide an anatomical foundation for subsequent studies rather than direct evidence supporting immediate clinical use of the prediction equation.

Previous studies evaluating anthropometric correlates of small bowel length have reported heterogeneous findings. Tacchino evaluated 443 surgical patients and found that height was the only independent anthropometric predictor of small bowel length, explaining approximately 12% of its variability [17]. Bekheit et al., in a larger cohort of 606 living adults, reported a mean TSBL of 630 ± 175 cm but found only very weak correlations between bowel length and both height and body weight, suggesting limited clinical predictive value of these variables when considered individually [8]. More recently, Sayadishahraki et al. evaluated 150 patients undergoing abdominal surgery and identified associations between small bowel length and several demographic and anthropometric characteristics, including height, although the magnitude and direction of some associations differed according to operative approach [10]. In comparison, the present study used a standardized autopsy-based anatomical protocol and a multivariable model incorporating height, weight, and waist circumference, which explained approximately 46% of TSBL variability. However, the RMSE of approximately 80 cm and the proportion of predictions within ±10% of observed TSBL of 56.0% indicate that the higher explained variance does not translate into sufficient individual-level precision for clinical decision-making. These differences across studies may reflect variation in study populations, bowel measurement conditions, anthropometric profiles, and modeling approaches, and further support the need for external validation before anthropometry-based prediction can be incorporated into surgical practice.

Several limitations should be considered when interpreting the findings of the present study. First, although the study included a relatively large autopsy cohort and employed a standardized measurement protocol, its single-center design and inclusion of cases from a Turkish forensic population may limit the generalizability of the results to populations with different demographic, ethnic, and anthropometric characteristics. In addition, the predominance of male cases (72.8%) may have influenced the distribution of anthropometric characteristics, although sex-stratified analyses and the sex × height interaction analysis did not indicate a substantial difference in the height–TSBL relationship between sexes. Race/skin color was not routinely recorded and therefore could not be evaluated as a potential source of anatomical variability. Second, despite the use of repeated in situ measurements and a reference ex situ assessment, bowel length measurement remains susceptible to methodological variability related to tissue handling, postmortem changes, and the absence of physiological bowel tone. Postmortem relaxation, loss of smooth-muscle tone, and changes in tissue hydration may result in differences between ex situ postmortem bowel length and bowel length measured intraoperatively in living patients. Although cases with advanced decomposition were excluded, the death-to-autopsy interval was restricted, and no significant association was identified between this interval and the principal anthropometric or anatomical measurements, these procedures cannot completely eliminate the potential effects of postmortem changes. Moreover, the study did not include a direct comparison between postmortem and in vivo bowel measurements. Third, although the three repeated in situ measurements showed high reproducibility, formal interobserver reliability testing of the reference ex situ measurement was not performed. Consequently, some degree of measurement variability related to the observer or tissue handling cannot be excluded. Fourth, the final prediction model explained approximately 45% of the variability in TSBL and had an RMSE of approximately 80 cm, with only 56.0% of predictions falling within ±10% of the observed value. These findings indicate substantial residual individual-level prediction error and limit the current use of the equation for precise operative decision-making. The model should therefore not be considered a replacement for direct intraoperative bowel measurement. Fifth, information regarding factors that may influence intestinal development, such as childhood nutritional status, genetic background, hormonal influences, and lifelong dietary habits, was not available. Imaging-derived anatomical variables and other biological determinants that might improve predictive accuracy were also not evaluated. Finally, the model underwent internal bootstrap validation only; no external validation or validation in a living bariatric surgical population was performed. Therefore, the proposed prediction equation should be considered exploratory until its performance is confirmed in independent cohorts. Despite these limitations, the large sample size, prospective data collection, standardized anatomical assessment, repeated in situ measurements, and internal validation procedures represent important strengths and provide valuable insights into the relationship between anthropometric characteristics and total small bowel length.

## 5. Conclusions

In conclusion, substantial interindividual variability in total small bowel length was observed in this large autopsy-based cohort. Among the anthropometric variables evaluated, body height emerged as the strongest independent predictor of bowel length, while body weight and waist circumference also contributed significantly to the observed variation. These findings suggest that readily obtainable anthropometric measurements may provide useful insights into underlying intestinal anatomy.

Furthermore, the proposed prediction model demonstrated moderate predictive performance but retained substantial individual-level prediction error, indicating that preoperative estimation of total small bowel length may be feasible only within limited precision. Accordingly, the model should currently be regarded as exploratory and as a potential source of supplementary preoperative information rather than as a clinically validated decision tool or a substitute for direct intraoperative bowel measurement.

Given the proportion of unexplained variability, the autopsy-based nature of the measurements, and the absence of in vivo and external validation, clinical implementation cannot yet be recommended. Future multicenter studies in living surgical populations, incorporating external validation, imaging-based variables, and additional biological or developmental determinants, are warranted to refine prediction accuracy and further clarify the role of anthropometry-based approaches in individualized surgical planning.

## Figures and Tables

**Table 1:** Baseline Demographic, Anthropometric, and Postmortem Characteristics of the Study Sample (*n* = 327).

Variable	Mean ± SD or *n* (%)
Age (years)	44.8 ± 16.7
Male sex	238 (72.8)
Female sex	89 (27.2)
Height (cm)	172.6 ± 8.9
Weight (kg)	79.4 ± 18.6
BMI (kg/m^2^)	26.5 ± 5.8
Waist circumference (cm)	94.1 ± 15.2
Abdominal fat thickness (cm)	3.8 ± 1.7
Total small bowel length, ex situ (cm)	612.4 ± 108.7
In situ measurement 1 (cm)	603.1 ± 106.4
In situ measurement 2 (cm)	606.5 ± 107.2
In situ measurement 3 (cm)	608.2 ± 106.8
Time from death to autopsy (hours)	18.7 ± 7.4
Cause of death	
Cardiovascular diseases	102 (31.2)
Trauma-related causes	81 (24.8)
Respiratory diseases	48 (14.7)
Intoxication	37 (11.3)
Neurological causes	29 (8.9)
Other causes	30 (9.1)

Data are presented as mean ± standard deviation or number (percentage). Abbreviations: BMI, body mass index; SD, standard deviation.

**Table 2:** Correlations Between Total Small Bowel Length and Anthropometric and Postmortem Variables.

Variable	Correlation Coefficient (r)	*p*-Value
Age (years)	−0.062	0.264
Height (cm)	0.612	<0.001
Weight (kg)	0.428	<0.001
BMI (kg/m^2^)	0.221	<0.001
Waist circumference (cm)	0.341	<0.001
Abdominal fat thickness (cm)	0.196	<0.001
Time from death to autopsy (hours)	0.041	0.460

Pearson correlation coefficients were calculated. Abbreviations: BMI, body mass index.

**Table 3:** Multivariable Linear Regression Analysis of Factors Associated With Total Small Bowel Length.

Variable	Unstandardized β (95% CI)	Standardized β	VIF	*p*-Value
Age (years)	−0.18 (−0.61 to 0.25)	−0.028	1.05	0.410
Male sex	12.4 (−10.2 to 35.0)	0.053	1.29	0.281
Height (cm)	5.46 (4.21 to 6.71)	0.447	1.46	<0.001
Weight (kg)	1.08 (0.31 to 1.85)	0.185	2.54	0.006
Waist circumference (cm)	0.71 (0.07 to 1.35)	0.099	2.27	0.029
Abdominal fat thickness (cm)	2.42 (−2.30 to 7.14)	0.038	1.43	0.314
**Model Statistic**		**Value**		
R^2^		0.461		
Adjusted R^2^		0.451		
F-statistic		45.6		
Model *p*-value		<0.001		

Total small bowel length was entered as the dependent variable. Abbreviations: CI, confidence interval; VIF, variance inflation factor.

**Table 4:** Development and Internal Validation of Models for Prediction of Total Small Bowel Length.

Model	Included Variables	R^2^	Adjusted R^2^	RMSE (cm)	MAE (cm)
Model 1	Height	0.374	0.372	85.9	67.7
Model 2	Height + Weight	0.439	0.436	81.3	64.1
Model 3 (final model)	Height + Weight + Waist circumference	0.456	0.451	80.1	62.8
**Internal Validation and Agreement Parameter**		**Value**			
Bootstrap optimism-corrected R^2^		0.444			
Optimism in adjusted R^2^		0.007			
Calibration slope		0.97			
Calibration intercept (cm)		18.4			
Mean prediction error/bias (cm)		0.2			
Lower 95% limit of agreement (cm)		−156.2			
Upper 95% limit of agreement (cm)		156.6			
Predictions within ±10% of observed TSBL		183/327 (56.0%)			

Internal validation was performed using 1000 bootstrap resamples. Abbreviations: MAE, mean absolute error; RMSE, root mean square error; TSBL, total small bowel length.

## Data Availability

The data that support the findings of this study are available from the corresponding author upon reasonable request. The data are not publicly available due to institutional and ethical restrictions.

## Abbreviations

BMI	Body Mass Index
CI	Confidence Interval
MAE	Mean Absolute Error
RMSE	Root Mean Square Error
R^2^	Coefficient of Determination
SD	Standard Deviation
TSBL	Total Small Bowel Length
VIF	Variance Inflation Factor
